# Hepatic Iron Overload and Hepatocellular Carcinoma: New Insights into Pathophysiological Mechanisms and Therapeutic Approaches

**DOI:** 10.3390/cancers17030392

**Published:** 2025-01-24

**Authors:** Elena Chatzikalil, Konstantinos Arvanitakis, Georgios Kalopitas, Matilda Florentin, Georgios Germanidis, Theocharis Koufakis, Elena E. Solomou

**Affiliations:** 1 Division of Pediatric Hematology-Oncology, First Department of Pediatrics, National and Kapodistrian University of Athens Medical School, 11527 Athens, Greece; elenachatz@med.uoa.gr; 2 “Aghia Sofia” Children’s Hospital ERN-PeadCan Center, 11527 Athens, Greece; 3 Division of Gastroenterology and Hepatology, First Department of Internal Medicine, AHEPA University Hospital, Aristotle University of Thessaloniki, St. Kiriakidi 1, 54636 Thessaloniki, Greece; arvanitak@auth.gr (K.A.); gekalopi@auth.gr (G.K.); geogerm@auth.gr (G.G.); 4 Basic and Translational Research Unit, Special Unit for Biomedical Research and Education, School of Medicine, Faculty of Health Sciences, Aristotle University of Thessaloniki, 54636 Thessaloniki, Greece; 5 Faculty of Medicine, School of Health Sciences, University of Ioannina, 45110 Ioannina, Greece; matildaflorentin@yahoo.com; 6 Second Propaedeutic Department of Internal Medicine, Hippokration General Hospital, Aristotle University of Thessaloniki, 54642 Thessaloniki, Greece; 7 Department of Internal Medicine, University of Patras Medical School, 26500 Rion, Greece

**Keywords:** hepatocellular carcinoma, MASLD, iron overload, HFE mutations, chelation therapy

## Abstract

Hepatocellular carcinoma (HCC), the most common form of primary liver cancer, is rising in global incidence and mortality. Metabolic dysfunction-associated steatotic liver disease (MASLD), one of the leading causes of chronic liver disease, is strongly linked to metabolic conditions that can progress to liver cirrhosis and HCC. Iron overload (IO), whether inherited or acquired, results in abnormal iron hepatic deposition, significantly impacting MASLD development and progression to HCC. While the pathophysiological connections between hepatic IO, MASLD, and HCC are not fully understood, dysregulation of glucose and lipid metabolism and IO-induced oxidative stress are being investigated as the primary drivers. Genomic analyses of inherited IO conditions reveal inconsistencies in the association of certain mutations with liver malignancies. Moreover, hepatic IO is also associated with hepcidin dysregulation and activation of ferroptosis, representing promising targets for HCC risk assessment and therapeutic intervention. Understanding the relationship between hepatic IO, MASLD, and HCC is essential for advancing clinical strategies against liver disease progression, particularly with recent IO-targeted therapies showing potential at improving liver biochemistry and insulin sensitivity. In this review, we summarize the current evidence on the pathophysiological association between hepatic IO and the progression of MASLD to HCC, underscoring the importance of early diagnosis, risk stratification, and targeted treatment for these interconnected conditions.

## Simple Summary

Hepatocellular carcinoma (HCC) is one of the most prevalent cancers globally, posing a substantial health burden. Ongoing research on primary HCC risk factors has revealed an epidemiological shift, with the focus moving from viral hepatitis to metabolic dysfunction-associated steatotic liver disease (MASLD). Recent studies on MASLD-related HCC risk factors have identified only a limited number of risk stratification tools and novel therapeutic options. Interestingly, iron metabolism appears to be dysregulated in both MASLD and HCC, with iron overload emerging as a sensitive diagnostic and prognostic marker for HCC. Therefore, the development of iron-oriented therapeutic strategies could play a critical role in HCC monitoring and treatment, contributing to global efforts that aim to improve overall survival of patients with HCC.

## 1. Introduction

HCC represents one of the most common neoplasms worldwide and has been among the most rapidly increasing types of cancer during the last decades [1]. Its mortality increased by 43% in the United States (from 7.2 to 10.3 deaths per 100,000 population) between 2000 and 2016 and its incidence-to-mortality ratio is the highest for any solid tumor, being 0.92 in the year 2023 [2,3]. HCC is also the second most lethal malignant neoplasm after pancreatic cancer with a 5-year survival-rate of 18%, despite the continuous research on novel therapeutic targets [4,5,6]. The main risk factors for the development of HCC include alcohol consumption (37%), hepatitis C virus (HCV) (31%), hepatitis B virus (HBV) (9%), and other non-viral, non-alcoholic liver conditions, currently under the term “metabolic dysfunction-associated steatotic liver disease” (MASLD) (23%) [7].

Metabolic dysfunction is characterized by having at least one of the following: obesity/overweight, type 2 diabetes mellitus (T2DM), hypertension, or dyslipidemia, while steatotic liver disease is diagnosed via liver biopsy and/or imaging [8]. However, the implementation of vaccination programs against HBV in newborns, the HCV treatment programs worldwide, and the increasing rates of obesity and metabolic syndrome in the developing and Western resource-rich countries have changed the epidemiology of HCC, which presents with a shift from viral hepatitis towards MASLD [2,7]. Screening and surveillance programs are not widely available for patients with non-cirrhotic MASLD, even though 30% of patients with MASLD-related HCC are not cirrhotic [7,9]. Considering that MASLD is expected to become the most common predisposing condition for HCC in Western countries, it is urgent for public health care policies to prioritize HCC surveillance in patients with MASLD, through the development of risk stratification models that will allow patients who are at the highest risk of developing HCC to receive the most effective screening [7].

Various studies have investigated the risk factors of MASLD-associated HCC, with T2DM, obesity, metabolic syndrome, smoking, altered gut microbiome, and genetic factors being the most common [9,10,11]. Despite extensive research in large cohorts aiming to identify well-established risk factors for HCC, few predictive models for effective risk stratification have been developed, and many aspects of hepatocarcinogenesis remain incompletely understood. Furthermore, studies on the pathophysiological mechanisms driving HCC have largely focused on genetic aspects, leading to the development of genetic risk scores. However, these scores present limited applicability in routine clinical settings, underscoring the need for more accessible and clinically relevant risk assessment tools [2,12]. The potential roles of various mechanisms that affect hepatocytes at both molecular and cellular level add another level of complexity on HCC risk stratification and on the application of screening methods [13,14,15]. Therefore, defining factors that can be included in novel risk stratification tools, including sex, age, race and ethnicity, body mass index, fasting triglycerides, alanine aminotransferase (ALT), fasting glucose, and blood pressure, is considered beneficial [3]. Interestingly, some authors report significant discrepancies in inflammatory markers of everyday clinical practice as potential diagnostic and prognostic markers for many types of cancer, including HCC. One promising marker is serum ferritin, which is indicative of iron overload [16].

Iron metabolism is disturbed in cases of MASLD and HCC with elevated serum ferritin being frequently observed, with or without elevated transferrin saturation [17]. In liver biopsy analyses, mild hepatic iron deposition has been demonstrated and is considered to be a factor exacerbating HCC development and progression [18]. Serum markers indicating iron overload have been investigated as basic components of HCC diagnostic and risk stratification tools in recent studies [19,20,21]. Iron deposition influences liver-related outcomes and predisposes individuals to extrahepatic disease complications. However, the precise pathophysiological mechanisms underlying the association between MASLD, HCC, and IO, as evidenced by hepatic iron deposition and/or elevated ferritin levels, remain incompletely understood. Factors such as dysregulated glucose and lipid metabolism, increased oxidative stress, and hereditary conditions associated with IO are among the primary contributors of IO in hepatocarcinogenesis. Additionally, recent advances in IO-targeted therapies have demonstrated promising outcomes, including reduction in liver enzyme levels and improvement of insulin sensitivity. In this review, we summarize current evidence on the pathophysiological link between IO and hepatocarcinogenesis, while also examining the potential role of IO in MASLD development. Furthermore, our objective is to provide information on novel diagnostic markers for HCC and to promote the integration of iron-targeted therapies into comprehensive treatment strategies for HCC.

## 2. Iron Metabolism and Iron Overload: An Overview of Pathophysiological Mechanisms and the Significance of Liver Involvement

### 2.1. A Brief Summary of Iron Metabolism

**Figure 1:**
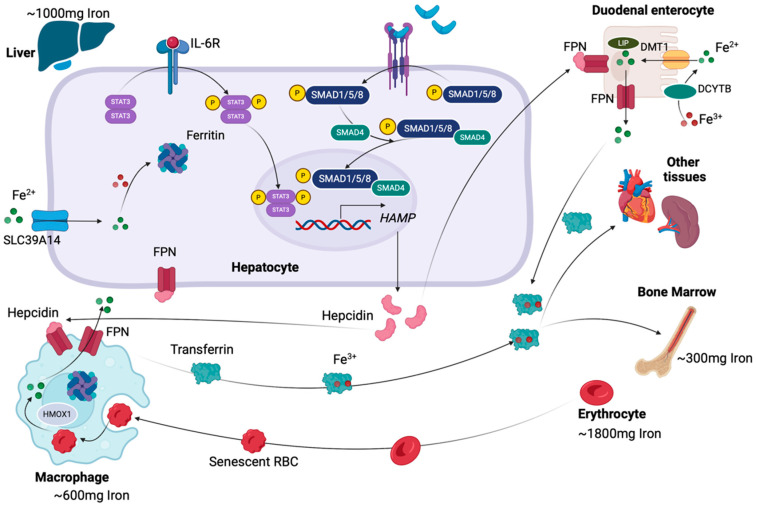
Schematic presentation of systemic iron metabolism. In plasma, iron circulates bound to transferrin, in Fe^3+^ valence state, and is distributed in red blood cells, liver, macrophages, bone marrow, and other tissues. The reduction of Fe^3+^ to Fe^2+^ ions is catalyzed by cytochrome b in the enterocytes. Iron’s transportation into the systemic circulation is controlled by ferroportin 1, with its expression being regulated by hepcidin. Hepcidin’s regulation is controlled by the BMP/SMAD pathway. BMP ligands downregulate the expression of cytoplasmic SMAD1, SMAD5, and SMAD8 proteins, which act synergistically with SMAD4, being translocated to the nucleus and blocking hepcidin expression. Small amounts of hepcidin are produced by activated macrophages, in response to inflammation. (The image was created using BioRender software version 04, License #*IL27OL0MQD*). [BMP, bone morphogenetic protein; DCYTB, duodenal cytochrome B; DMT1, divalent metal transporter 1; Hamp, hepcidin antimicrobial peptide gene; HMOX1, heme oxygenase 1; IL-6R, interleukin-6 receptor; FPN, ferroportin; LIP; labile iron pool; RBC, red blood cell; SLC39A14, solute carrier family 39 member 14; SMAD, sons of mothers against decapentaplegic homologue; STAT3, signal transducer and activator of transcription 3].

Iron, a d-block transition metal, is the most abundant metal on earth and one of the most crucial catalysts of the first reactions of human life [22]. The most common iron states are the divalent ferrous (Fe^2+^) and the trivalent ferric (Fe^3+^) [23]. Iron’s ability to interchange between Fe^2+^ and Fe^3+^ valence states (the Fenton reaction) enhances its participation in various cellular processes, including electron transport and ATP generation, gene regulation, host defense, and nucleic acid replication and repair [16]. Iron bound to protein side chains is used by at least 2% of human proteins, specifically within the porphyrin ring of heme in hemoproteins, or within iron–sulfur (Fe–S) clusters, in non-heme iron-containing proteins [16]. The average iron concentration in a normal adult is 50–60 mg of iron per kg of body weight, which is distributed in red blood cells, liver, macrophages, bone marrow, and other tissues [24]. Hemoproteins include hemoglobin, which contains the vast majority (approximately 65%, ~1800 mg) of iron in the body, and myoglobin, which are responsible for oxygen storage and transport, catalase and peroxidase enzymes, which are involved in primary oxygen metabolism, and cytochromes, which take part in redox reactions and electron transport [22,23]. Myoglobin, enzymes, and cytochromes contain approximately 10% of the total iron, while 12.5% is found in macrophages of the reticuloendothelial system (~600 mg), 10% in hepatocytes (~1000 mg), and 4% in the bone marrow (~300 mg) [22,25]. (Figure 1). Non-heme iron-containing proteins also have various crucial functions, being involved in DNA synthesis, cell proliferation and differentiation, gene regulation, drug metabolism, and steroid synthesis [22,23].

Human cells acquire iron through different mechanisms, including receptor-mediated endocytosis, iron bound complexes, iron ions, or iron chelates through solute carriers and phagocytosis of other cells [25]. In plasma, iron circulates mostly bound to transferrin, a single-chain glycoprotein with high affinity with the Fe^3+^ valence state, which transfers iron molecules systemically, to bone marrow, and to other organs [26]. Transferrin is mainly derived from hepatocytes, with extra-hepatic sources partially compensating in cases of lack of liver transferrin [26]. Iron absorption occurs mainly within the gastrointestinal tract, which could be explained due to the more acidic environment in the duodenum and proximal jejunum [27]. The enzyme which catalyzes the reduction of Fe^3+^ to Fe^2+^ ions, termed duodenal cytochrome b (Dcytb), is localized on enterocytes. Fe^2+^ ions are transported into the duodenal enterocytes via the divalent metal transporter 1 (DMT1), by a proton (H^+^)-coupled process, with the involvement of another brush-border membrane transporter, the sodium/hydrogen exchanger (NHE), which allows proton recycling across the duodenal luminal membrane [23]. After being absorbed by the enterocytes, iron joins the intracellular labile iron pool (LIP) [28]. In cases of low demand for iron, the absorbed iron is stored within the hepatocytes in the form of ferritin [16]. Iron enters the hepatocytes via SLC39A14 transporter [23]. Ferritin is a 24-subunit protein, with an icosahedral cage-like structure and a molecular weight of ~450 kDa, consisting of two types of subunits (H subunit, which is the heavier, and L subunit, which is lighter), and is the major protein for iron storage and detoxification [29]. During iron release, ferritin produces free radicals, which are involved in many physiological processes, including phosphoprotein inactivation, lipid peroxidation, and aging [29]. The enterocyte’s life span is short since they are desquamated and replaced every few days, and, as a result, iron stored in ferritin is lost upon enterocyte destruction [23]. In cases of high iron demand, the absorbed iron is transported across the basolateral membrane into the blood stream [26], and after being absorbed and released into the circulation, iron is bound on transferrin, which binds with up to two iron molecules [23]. By this double binding, iron is delivered through receptor-mediated endocytosis [23].

Iron transport into the systemic circulation is controlled by ferroportin 1 (FPN1), whose expression is regulated by the hepatic hormone hepcidin [30]. Hepcidin is an antimicrobial peptide, consisting of 25 amino acids, containing high amounts of cysteine, and being stabilized by four disulfide bonds. Hepcidin is the master regulator of systemic iron homeostasis [31]. Its expression is regulated by serum and liver iron levels, as well as by erythropoietic activity and inflammatory conditions [31]. Hepcidin, after being released from hepatocytes, is bound to FPN1, and regulates serum iron levels by enhancing internalization, ubiquitination, and degradation of the ligand/receptor complex [30]. Hepcidin regulation by iron is directly controlled by the iron-induced bone morphogenic protein (BMP)/SMAD pathway [31]. BMP ligands bind to type I and type II serine threonine kinase receptors downregulating the expression of cytoplasmic SMAD1, SMAD5, and SMAD8 proteins, which act synergistically with SMAD4, being translocated to the nucleus and ultimately downregulating hepcidin expression. Moreover, hepatocytes have the ability to sense iron levels by the expression of TFR1, TFR2, and HFE [30,32]. In cases of low serum iron levels, HFE binds with TFR1, while in cases of increased iron levels, TFR1 expression is downregulated and TFR2 expression is upregulated, and as a result, TFR2 stability is increased and the HFE binding to TFR2 is enhanced [30,32]. Furthermore, the HFE/TFR2 complex interacts with an iron-specific BMP co-receptor, termed hemojuvelin (HJV), resulting in dysregulated expression of hepcidin, while matriptase-2, a membrane serine protease encoded by the gene *TMPRSS6*, has been demonstrated to release HJV from hepatocytes lowering the ability of HJV to act as a co-receptor and inhibiting HAMP (hepcidin gene) expression [32,33]. Finally, in cases of increased hepcidin levels, iron absorption by the enterocytes and iron recycling in macrophages are both inhibited, while activated macrophages produce small amounts of hepcidin in response to inflammation, inhibiting iron export via autocrine processes. Iron regulation, export, transportation, and storage are summarized in Figure 1.

### 2.2. Iron Overload and Its Role in Liver Damage

**Figure 2:**
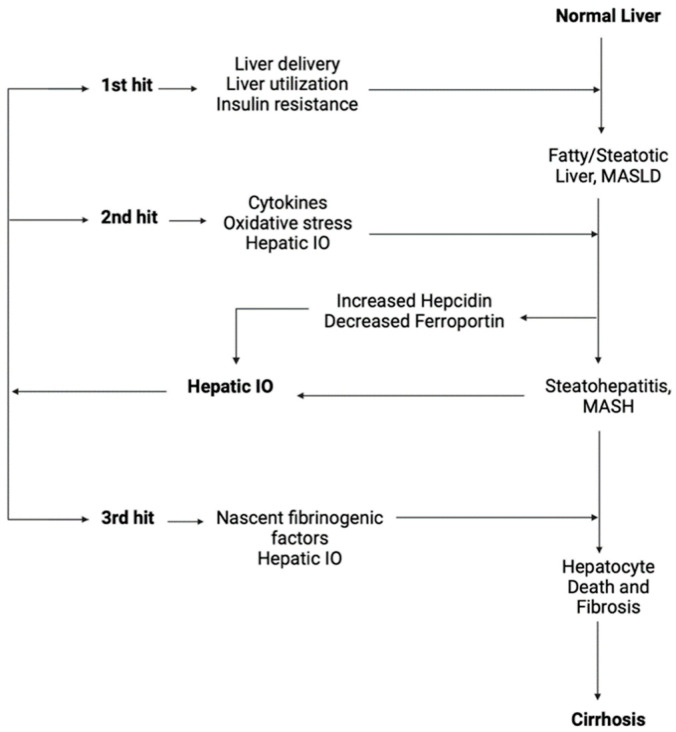
Schematic diagram illustrating disease progression of metabolic dysfunction-associated steatohepatitis. IO plays a role in the progression of cirrhosis by generating the second hit. In MASLD, IO promotes the development of steatohepatitis by a “feed-forward” mechanism, generating the third hit, liver injury, fibrosis, and cirrhosis, predisposing to HCC development. (The image was created using BioRender software version 04, License #*YW27OL0RWV*). [IO: iron overload; MASLD: metabolic dysfunction-associated liver disease; MASH: metabolic dysfunction-associated steatohepatitis].

Disruptions in the BMP/SMAD signaling pathway downregulate hepcidin levels resulting in IO, which is defined by an increase of 5 g or more of the total iron (average normal levels: 2–4 g) of the human body [34]. This condition dysregulates the normal activity of storage proteins and large amounts of iron are released into the cytoplasm of liver cells and, as a result, liver is the main target organ for iron deposition and overload [35]. In cases of IO, the body releases proteins that affect FPN1 expression in order to reduce the excess iron [20]. The main constituent of these protein products is hepcidin, which regulates plasma iron levels by preventing intestinal iron absorption and macrophage iron circulation [31]. During the last two decades, it has been proven that IO may predispose to HCC [35]. Hereditary hemochromatosis was the first condition of IO which was investigated in the basis of this predisposition [36]. The exact mechanisms by which hereditary hemochromatosis protein (HFE) and TFR2 regulate iron homeostasis are still under investigation; however, there is evidence suggesting that HFE and TFR2 interact with HJV, mostly in cases with high transferrin saturation, enhancing HJV-induced hepcidin expression [37]. Iron accumulates in the liver not only in congenital systemic iron-loading conditions, but also in various other ways, including systemic macrophage iron accumulation due to systematic transfusions or conditions of hemolysis, chronic hepatitis (e.g., hepatitis C, alcoholic liver disease, porphyria cutanea tarda), and liver-specific iron accumulation in cirrhosis [38]. Interestingly, HCC has been diagnosed in cases of IO in the absence of cirrhosis [39], but the mechanism of pathogenesis of HCC in cirrhotic patients with IO is more well-defined (Figure 2) [40].

Currently, a three-hit hypothesis explaining the mechanism between IO and the progress of liver disease has been proposed, suggesting that IO may play a role in the development of MASLD-associated HCC (Figure 2) [40]. Steatosis, cytokines, and reactive oxygen species (ROS) secondary to IO propagate oxidative stress (OS) and inflammation, forming a favorable environment for the third hit: hepatic injury, overwhelming the ability of hepatic regeneration [40]. These conditions of liver dysfunction result in dysregulated expression of proteins participating in iron metabolism. During the process of hepatocyte regeneration, the expression of transferrin receptor may be increased, contributing further to IO [40]. Moreover, altered levels of ferroportin and hepcidin are observed. In more detail, a reduced expression of ferroportin and HJV protein has been described in patients with liver damage and has been associated with increased TNF-α expression, increased iron accumulation, and upregulated hepcidin expression, aggravating liver dysfunction [40,41]. IO has been observed in patients with MASLD and is considered to affect lipid and glucose metabolism, leading to insulin resistance [42]. Additionally, IO promotes ROS production and fibrinogenesis, contributing to the development and progression of both MASLD and HCC [36,42].

The first indication of IO is high serum ferritin levels, while ferritin is a potential marker for cancer prognosis. High serum ferritin levels have been demonstrated in numerous neoplasms, including lung cancer, pancreatic cancer, endometrial carcinoma, hepatobiliary tumors, cervical cancer, testicular germ cell tumors, and hematologic malignancies, namely acute myeloid leukemia, Hodgkin lymphoma, T-cell lymphoma, and multiple myeloma [43]. Serum ferritin is considered to have predictive and prognostic value of malignant disease, being a marker of treatment sensitivity and toxicity in chemotherapy, as well as an indicator of tumor downstaging, in cases of surgical resection [44,45]. Serum ferritin levels have been demonstrated to decrease weeks after chemotherapy induction, increase in cases of toxicity in platinum-based chemotherapy, and return to normal levels after surgery, redefining risk stratification in solid tumors [46]. Real-world data also suggest that serum ferritin is elevated in HCC and that high ferritin levels are associated with poor overall survival and progression-free survival [43,47]. Specifically, in a 10-year cohort study, serum ferritin was suggested as an independent prognostic marker for the overall survival of patients with advanced hepatobiliary cancer [19]. In another study on the impact of preoperative serum ferritin levels in patients with HCC undergoing TACE, preoperative serum ferritin was negatively correlated with overall survival and progression-free survival of patients [47]. Notably, a prognostic ratio, termed the ferritin/globulin ratio, was recently proposed as an indicator of hepatocarcinogenesis, being significantly higher in HCC patients than in healthy controls [19]. Moreover, in a cohort of MASLD patients without major underlying causes of chronic liver disease, increased levels of serum iron and transferrin saturation were significantly associated with elevated risk of hepatocarcinogenesis, suggesting that clinical surveillance of serum iron levels could be a potential diagnostic strategy for identifying MASLD patients who are more likely to develop HCC [20]. Additionally, a survival analysis of 427 HCC patients who underwent radical hepatectomy revealed a significant negative correlation between preoperative serum ferritin and patients’ survival, proposing preoperative serum ferritin levels as a convenient and reliable predictor of survival outcomes of HCC patients after radical hepatic resection [21].

## 3. The Role of IO-Induced Oxidative Stress in Hepatocarcinogenesis

### 3.1. Oxidative Stress: Correlation with Iron Metabolism and Associated Liver Disease Pathogenesis

OS is a result of an imbalance between ROS production and accumulation, due to harmful endogenous and exogenous factors, including IO [48]. Free radicals, mainly ROS and reactive nitrogen species (RNS), which represent unstable high reactive metabolites in several redox reactions during normal cellular metabolism, are upregulated in OS states [49]. IO has been demonstrated to generate OS by interchanging between Fe^2+^ and Fe^3+^ valence states through the Fenton reaction, increasing the steady state concentration of ROS and RNS [48]. During this process, H_2_O_2_ presents with a greater stability, as compared to O^2−^ [50]. In the Fenton reaction, H_2_O_2_ is catalyzed to produce hydroxyl radicals (OH.), which are the most reactive and harmful ROS. The toxicity of O^2−^ and H_2_O_2_ arises from their iron-dependent conversion into the extremely reactive hydroxyl radical OH. (the Haber–Weiss reaction), which causes severe damage to cell organelles, mainly membranes, proteins, and DNA [51].

The progression from MASLD to MASH is accompanied by mitochondrial ROS and oxidative mitochondrial DNA damage, while also by iron accumulation and deposition within the hepatocytes and an upregulation of serum ferritin, further propagating ROS formation [51,52]. During the process of MASLD development and progression, OS induces insulin resistance, inflammation, lipotoxicity, and stellate cell activation [53,54]. ROS excess contributes to the progression from MASLD to MASH. In a study on 152 patients with MASLD, severe/advanced disease was associated with increased lipid peroxidation, correlating oxidative damage with MASLD progression [55], while another animal study reported a significant relationship between lipid peroxidation, expression of antioxidant genes, increased hepatic OS, and severe MASH histology [56]. Another study has also highlighted the dual role of ROS in HCC, as elevated ROS enhanced hepatocyte cytotoxicity and apoptosis [50]. ROS mainly stimulate the accumulation of DNA mutations, inducing MASH progression into HCC [57]. Four aspects are involved in OS-associated hepatocarcinogenesis, inducing HCC cell proliferation, invasion, and metastasis: genetic alterations, signaling pathway modifications, transcription factor dysregulation, and TME alterations [51].

### 3.2. OS-Associated Hepatocarcinogenesis: The Four Major Aspects

Genetic and epigenetic alterations, including oxidative nuclear and mitochondrial DNA damage, DNA hypomethylation, and alterations in microRNA expression, are associated with increased OS, playing a crucial role in HCC development and progression [58]. Due to lack of histone protection, mitochondrial DNA is prone to OS-related damage [59]. A recent animal study provided evidence that mitochondrial ROS levels increased by 200% during disease progression of nitrosodiethylamine-induced HCC, leading to proto-oncogene activation, promoting hepatocarcinogenesis [60,61]. Moreover, Xie et al., while investigating the hepatitis B virus x gene in the human liver cell line HL7702, reported an overactivated NLRP3 inflammasome in HL7702 cells, alongside increased mitochondrial membrane permeability, and upregulated ROS generation [62]. Regarding HCV virus, it inhibits the production of ROS through electron transport chain and induces mitochondrial damage and ROS formation by enhancing CYP2E1 expression [63,64]. DNA methylation is affected by OS in cases of IO, due to expression changes in certain enzymes, specifically histone methylases, and histone deacetylases (HDACs), which are triggered by induced Snail expression, which is associated with hypermethylation states via transporting HDAC1 and DNMT1 to sites of Snail occupancy [65]. ROS induction, Snail upregulation, E-cadherin downregulation, E-cadherin promoter hypermethylation, and increased Forkhead box C1 (FOXC1) expression via the ERK1/2-pELK1 pathway are epigenetic alterations associated with excess IO-induced hepatic ROS [65,66]. Micro RNAs (miRNAs) are major regulators of oncogenes and tumor suppressor genes in HCC, being correlated with epigenetics, inflammation, viral infection, and oxidative stress [67]. MiRNA overexpression and telomerase activity activation are associated with the accumulation of ROS-mediated oxidative DNA damage regulated by IO during hepatocarcinogenesis [68].

Moreover, OS modifies the expression of several signaling pathways involved in HCC pathogenesis, mainly the Wnt/β-catenin, PI3K/AKT/mTOR, and Notch pathways [51]. The Wnt/β-catenin pathway affects the metastatic potential and treatment resistance of HCC [69]. ROS stimulate the Wnt pathway, promoting hepatocarcinogenesis via the modulation of phosphorylation, proteasomal degradation, and T-cell activity [70]. A destruction complex, consisting of Axin, APC, casein kinase 1, and glycogen synthase kinase 3β, modulates the expression of cytoplasmic β-catenin [71]. In absence of Wnt ligands, the destruction complex binds to and phosphorylates β-catenin, which is recognized by the E3 ubiquitin ligase β-transducing repeats-containing protein (β-TRCP) [71]. These processes are followed by proteasomal degradation of β-catenin and inhibition of the Wnt/β-catenin pathway [72,73]. Phosphorylation of glycogen synthase kinase 3β is upregulated by ROS production leading to the inactivation of glycogen synthase kinase 3β via stimulation of the PI3K/AKT signaling pathway [71]. The PI3K/AKT/mTOR pathway is altered in HCC due to dysregulation of receptor tyrosine kinases (RTKs), which activate the lipid kinase PI3K, catalyzing phosphorylation of phosphatidylinositol [74]. Specifically, activated PI3K phosphorylates phosphatidylinositol-4,5-bisphosphate creating phosphatidylinositol-3,4,5-triphosphate, which interacts with phosphoinositide-dependent protein kinase 1 (PDK1) and Akt [74,75]. The tumor suppressor phosphate and tensin homolog (PTEN) catalyzes dephosphorylation of phosphatidylinositol-3,4,5-triphosphate to generate phosphatidylinositol-4,5-bisphosphate, which also controls Akt signaling [74,75]. MTOR complex 1 and mTOR complex 2 are created due to protein-binding and activation of mTOR [76]. Stimulation of the PI3K/Akt/mTOR signaling pathway is associated with HCC cell proliferation, migration, invasion, and drug resistance [77]. ROS regulates apoptotic activity by inhibiting the PI3K/Akt/mTOR signaling pathway, which partially controls ROS concentration, suggesting a dual function for oxidative stress in cancer. In addition, ROS regulates telomerase activity through activation of Akt signaling, enhancing hepatocarcinogenesis [78,79]. Furthermore, considering the negative correlation between Wnt/β-catenin signaling and EMT, inhibiting the Wnt pathway by ROS production can make HCC cells more sensitive to chemotherapy [51]. Last but not least, the Notch signaling pathway is associated both with steatotic and highly oxidative hepatic states and is partially modulated by overproduction of H_2_O_2_, while it also suppresses tumor progression and induces angiogenesis dysregulation [80]. ROS stimulate Notch signaling activation, enhancing epithelial-mesenchymal transition and HCC metastatic capacity [81,82].

IO-induced ROS stimulates several transcription factors, including forkhead box O (FOXO), hypoxia-inducible factor-1 alpha (HIF-1α), heat shock factor 1 (HSF1), NF-κB, and p53, while the activation of these molecules subsequently regulates the cellular redox state [83]. FOXO transcription factors present with a dual role in regulating hepatocarcinogenesis, either by promoting or suppressing tumorigenic signals. In more detail, FOXO3, via ROS-induced hepatotoxins and a positive feedback loop between Akt and mTORC2, promotes the development of HCC by indirectly inducing the transcription of the pentose phosphate pathway [84]. HIF-1α is commonly expressed in HCC and is triggered by IO-induced (as well as non-IO-induced) ROS generation, promoting the transcription of genes that improve glycolysis within an anaerobic environment [85]. HSF1 specifically responds to ROS through induction of genes encoding heat shock protein chaperones, and specifically via oxidation of internal Cys-35 and Cys-105 residues, and subsequent stimulation of antioxidant gene expression [86,87]. In HSF1-depleted HCC cell lines, glucose intake, lactate generation rates, and intercellular ROS levels were found to be decreased [88]. These observations indicate a positive correlation between ROS levels and HSF1 expression and suggest that excess ROS and upregulated HSF-1 promote hepatocarcinogenesis [89]. NF-κB modulates an adaptive immune response regulating the expression of antioxidant genes and facilitating HCC cell survival, growth, and metastasis [90]. In HCC, ROS produced in IO states activate NF-κB, promoting hepatocarcinogenesis and invasion and metastasis of HCC [91], while p53 induces apoptosis of HCC cells and inhibits cell proliferation [92].

The TME alterations represent another important aspect of ROS-regulated hepatocarcinogenesis. The dysregulated iron metabolism in HCC TME affects the phenotype of TME cells, mainly of the innate immune cells [93]. Specifically, HCC cells equipped with excessive iron uptake and decreased iron export machineries, deprive HCC TME of iron, and overproduce iron-related byproducts, including ROS enhancing oncogenic capacity or suppressing the antitumor activity of innate immunity [93]. OS affects several TME elements, mainly tumor-associated macrophages (TAMs), neutrophils, myeloid-derived suppressor cells (MDSCs), and Treg cells within the TME [51]. TAMs within the TME are involved in tumorigenesis via a variety of factors, including interleukin-10 (IL-10), tumor growth factor β (TGF-β), epidermal growth factor (EGF), and chemokines (CXCL17 and CCL24) [94]. ROS partially control macrophage differentiation, regulating tumorigenesis [51], and TAMs promote tumorigenesis via proangiogenic and immune-suppressive functions, while ROS production is critical for macrophage differentiation and blockage by the inhibition of superoxide anion (O^2−^) production [95]. It has been suggested that during the process of monocyte differentiation, O^2−^ is generated and a biphasic ERK signaling pathway is activated, resulting in TAM differentiation, while ROS-related inhibitors (e.g., butylated hydroxyanisole) inhibit macrophage differentiation, which in vitro suppresses tumorigenesis [95]. Targeting TAMs by blocking ROS has been proposed as an effective option for cancer treatment [95]. Moreover, proinflammatory cytokines and Toll-like receptor (TLR) agonists within the HCC TME may regulate several signaling pathways’ (specifically, Stat1, NF-κB, and C/EBPβ) activation, leading in upregulation of hepcidin and inhibition of ferroportin [93]. The upregulation of the Jak/Stat3 signaling pathway, which is activated by interleukin-10, is positively correlated with the expression of lipocalin-2 in macrophages [96]. Lipocalin-2 is an iron regulatory protein released from macrophages in the HCC TME, which can bind to its receptor in HCC cells and in macrophages regulating tumor cell growth and M2 polarization, respectively, and further inducing VEGF expression, enhancing lymphangiogenesis and cancer metastasis [96]. Furthermore, neutrophils are considered key mediators of the immunosuppressive environment, enhancing HCC progression; ROS-related neutrophil-regulating proteins may increase tumor cells migrative and invasive capacity [95]. Granulocyte-macrophage colony-stimulating factor (GM-CSF), produced by metastatic HCC cells, induces the activation of the Jak/Stat5β signaling pathway and transferrin synthesis in neutrophils, while lipocalin-2 released by neutrophils induces the activation of Src family kinases (SFKs), enhancing tumorigenesis [93,97]. S100A9, which belongs to a class of proteins termed damage-associated molecular patterns (DAMPs), is upregulated by excess ROS, and has proven in vitro to affect neutrophil recruitment in acute and chronic hepatic damage, by inducing neutrophil stimulation and degranulation [98]. S100A9, which is also regulated by HBV, indirectly promotes the proliferation of neutrophil extracellular traps, and activates neutrophils [98].

## 4. Dysregulated Glucose and Lipid Metabolism in Iron Overload States

### 4.1. Iron Overload-Induced Insulin Resistance and Its Association with Fibrotic Changes: From MASLD to HCC

There are numerous studies demonstrating that metabolic disorders, including obesity, insulin resistance, T2DM, polycystic ovary syndrome, and hypertriglyceridemia, are closely associated with MASLD; therefore, MASLD is considered to be the hepatic manifestation of metabolic syndrome [99]. Impaired glucose metabolism is a risk factor for MASLD, which has been associated with the development of HCC [100]. Interestingly, iron metabolism is involved in the pathogenesis of insulin resistance, and also in glucose homeostasis, via complex regulatory mechanisms, both at molecular and cellular level [84]. Several studies have reported a tight correlation between elevated serum ferritin, the first indication of IO, and insulin resistance, obesity, metabolic syndrome, and T2DM [101]. Transferrin, which is responsible for iron systemic transport, is associated with insulin resistance; elevated transferrin levels have been observed in many cohorts with obesity and chronic inflammatory state, predisposing to insulin resistance. Obesity and inflammation may cause pathological alterations in iron metabolism and transferrin function, which can eventually contribute to the dysregulation of insulin sensitivity [102,103].

**Figure 3:**
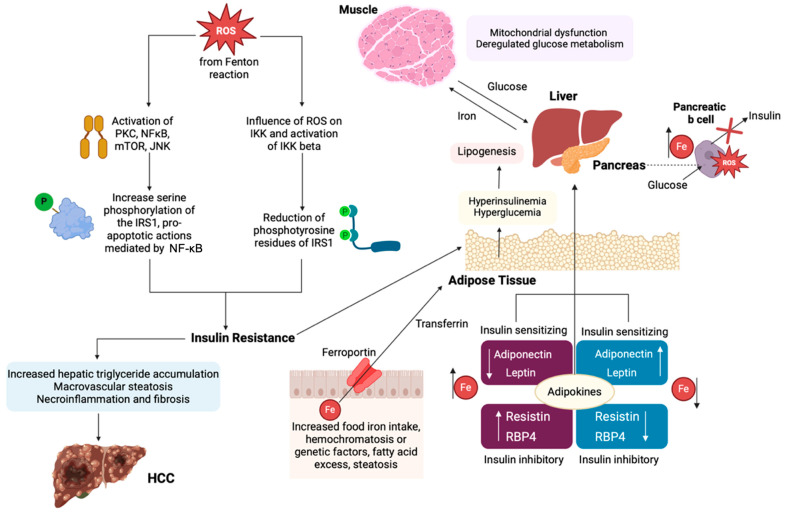
Pathogenetic mechanisms of iron-induced insulin resistance and its involvement in hepatocarcinogenesis. (The image was created using BioRender software version 04, License #*AB27OL2R2W*). [Fe: iron; JNK: c-Jun-N-terminal kinase; HCC: hepatocellular carcinoma; mTOR: mammalian target of rapamycin; NF-κB: nuclear factor κB; PKC: protein kinase c; RBP4: retinol binding protein 4; ROS: reactive oxygen species].

Iron metabolism plays a crucial role in the pathogenesis of insulin resistance, as well as in the dysregulation of glucose homeostasis, via complex regulatory mechanisms, both at a molecular and cellular level. The key aspect of this association is iron’s regulatory role in the function of the insulin receptor, and specifically in the modulation of insulin receptor tyrosine phosphorylation, which influences kinase activity down streaming signaling cascades, regulating insulin sensitivity at the cellular membrane [104]. Moreover, iron is involved in cellular processes, including ROS production and the inflammatory cascade, subsequently affecting insulin production [104]. Excess iron, by catalyzing ROS through the Fenton reaction, enhances the OS that interferes with insulin signaling cascades (mTOR, NF-κB, PKC, JNK activation, IRS1 tyrosine reduction, increased serine phosphorylation), resulting in insulin resistance [104]. The inhibition of mitochondrial superoxide dismutase as a result of IO-related mitochondrial dysfunction caused by oxidative damage, as well as the activation of NF-κB in macrophages and Kupffer cells and the release of TNFα, are crucial factors downregulating insulin signaling and decreasing adiponectin levels, being involved in the pathogenesis of insulin resistance (Figure 3 [105]. Furthermore, iron regulates transcription factors of glucose metabolism [90], while proteins involved in iron metabolism regulate cellular energy metabolism, affecting insulin sensitivity. Furthermore, the inflammatory state associated with metabolic syndrome modulates iron metabolism and dysregulation of iron, in turn, modulates immune responses, creating a feedback loop that improves chronic insulin resistance and disrupts insulin-mediated glucose uptake [104].

The accumulation of iron by adipocytes is an important factor of insulin resistance, considering that iron is a key modulator of the synthesis of insulin-regulating adipokines [106]. Specifically, iron accumulation enhances the expression of resistin and retinol-binding protein 4 (RBP-4), while it inhibits the expression of insulin sensitizing leptin, resulting in insulin resistance (Figure 3) [106]. In vitro, iron downregulates the expression of insulin-sensitizing leptin by the inactivation of CREB, via a phosphorylation-dependent mechanism [107]. However, in an animal model of hereditary hemochromatosis, results demonstrated increased adiponectin expression and improved glucose tolerance, which were attributed to reduced iron content of adipose tissue, in spite of systemic IO [108]. Nevertheless, further experiments with adipocyte-specific ferroportin knockout mice could clarify the role of IO-induced insulin resistance to hepatocarcinogenesis [108]. Additionally, given the importance of iron metabolism in pancreatic β cell function, excessive iron accumulation is expected to impair insulin secretion, especially in IO-induced oxidative stress (Figure 3) [109]. In an animal study, DMT1 was identified as an important mediator of IO and pancreatic β cell dysfunction, after its induction by the inflammatory cytokine IL-1β, demonstrating defects in glucose-stimulated insulin secretion in the absence of inflammatory stimulation, highlighting the importance of iron homeostasis in proper pancreatic β cell function, as well as the role of DMT1 in β cell iron supply [110].

### 4.2. Interactions Between Liver Iron and Lipid Metabolism and Their Implication in MASLD and HCC

The liver, apart from being the main site for iron storage, is a major regulator of lipid metabolism. Hepatic triglyceride (TG) synthesis uses fatty acids derived from plasma non-esterified fatty acids (NEFAs) and free fatty acids (FFAs), that are formed through de novo lipogenesis (DNL) within the liver [111]. The hepatic uptake of FFAs from plasma is directly correlated with the concentration of the NEFA pool, while TG lipolysis is considered to be the most important contributor to plasma NEFA [111]. FFAs, after being obtained by the liver via FFA binding protein and FFA translocase, are oxidated or detoxified by re-esterification with glycerol and cholesterol, producing TG and cholesteryl esters (CEs), respectively [111]. The TG and CE are secreted in the form of very-low-density lipoprotein (VLDL) into the circulation or are stored in the cytoplasm of hepatocytes in the form of lipid droplets [112]. Furthermore, insulin is a fat-sparing hormone and one of the main regulators of lipid metabolism [111]. In more detail, insulin increases TG synthesis by hepatocytes and downregulates its secretion by promoting the excess newly synthesized TG into cytosolic stores [113]. Moreover, insulin hinders lipolysis within the hepatocytes, decreasing VLDL by impairing the connection between apo-B100 and TG, and, furthermore, by stimulating the degradation of apo-B, which decreases VLDL-TG secretion [111]. It is thus evident that lipid metabolism and insulin are tightly correlated, interacting with iron metabolism and being affected in cases of IO.

Iron affects both directly and indirectly the lipid metabolic processes. Iron induces OS and ROS production via the Fenton reaction, and at the same time, it enhances inflammation, lipid peroxidation, fatty acid profile modification, and cell membrane damage [114]. ROS also stimulate hepatic stellate cells to increase collagen production, which results in the progression of fibrosis, and it also changes the ratio of saturated to unsaturated membrane phospholipids, dysregulating membrane fluidity [114]. Furthermore, peroxidation products of hepatocytes increase polyunsaturated fatty acids, which in cases of IO, inhibit lipogenic genes (e.g., FAS) by producing peroxidative cytotoxic effects [115]. Moreover, iron has a direct effect on lipid metabolism, as it modulates lipid storage and lipid secretion [111]. A characteristic example is iron deficiency anemia, which has been associated with increased hepatic lipogenesis and lipemia [111]. Regarding IO, its direct association with lipid metabolism has been studied mainly in vitro, with many recent studies investigating the direct effects of IO on lipid metabolism and providing various results [116]. In an animal study on mouse models with dietary IO, an increase in the activity of acyl-CoA cholesterol acyltransferase (ACAT) and a decrease in HMG CoA reductase and 7 α-hydroxylase were observed, and they were both correlated with hypercholesterolemia and unaltered hepatic cholesterol content, indicating that cholesterol’s synthetic and excretory pathways are not affected by IO, while the secretory pathway may be upregulated, resulting in hypercholesterolemia [112]. Another study however, demonstrated that transcripts of seven enzymes, including HMG CoA reductase, increased significantly in cases of IO, suggesting that hepatic iron excess upregulates the cholesterol synthetic pathway [116]. In addition, in vitro results showed that IO increases intracellular lipid droplet accumulation by an upregulated expression of a major histocompatibility complex class 1 molecule [117]. HCC cells rely heavily on FFAs to achieve cell membrane function, signaling molecular activity, and energy reserves for proliferation and metastatic processes [118]. HCC is associated with a dysregulated lipid metabolism, consisting of enhanced lipid synthesis and uptake, while fatty acid metabolism is suggested to play an important role in the metabolic reprogramming of the HCC TME [118]. The enhanced lipid synthesis and the increase in lipid droplets observed in IO contribute to the progression and metastatic potential of HCC [118].

**Figure 4:**
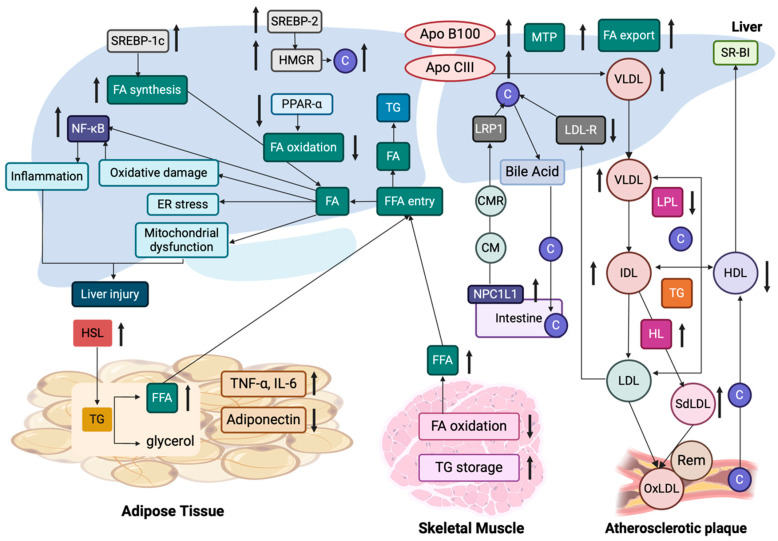
An illustration of the dysregulated lipid metabolism mediated by insulin resistance alongside its association with the development of liver injury, predisposing to MASLD and HCC development and progression. (The image was created using BioRender software version 04, License #*TN27OL0YNJ*). [CM: chylomicron; FFA: free fatty acid; HDL: high-density lipoprotein cholesterol; HMGR: 3-hydroxy-3-methyl-glutaryl-CoA reductase; HL: hepatic lipase; LDL: low-density lipoprotein; LPL: lipoprotein lipase; LRP1: LDL receptor-related protein 1; MTP: microsomal TG transfer protein; NF-κB: nuclear factor-κB; OxLDL: oxidized LDL; PPAR: peroxisome proliferator-activated receptor; SREBP: sterol regulatory element binding protein; TNF-α: tumor necrosis factor-alpha; TG: triglyceride; VLDL: very low-density lipoprotein].

High serum TG levels and low serum high-density lipoprotein (HDL) are observed in patients with MASLD [119]. The prevalence of MASLD in patients with dyslipidemia is estimated at approximately 50% [119]. A recent study suggested that peripheral insulin resistance is correlated with the pathogenesis of MASLD by a hyper-insulinemic euglycemic clamp [120]. Interestingly, abnormalities of lipid metabolism induced by insulin resistance highly affect MASLD via several pathways (Figure 4). Factors associated with adipose tissue, including tumor necrosis factor alpha (TNF-α), interleukin-6 (IL-6), and IL-1b, are linked to systemic insulin resistance. Lipoprotein lipase (LPL) hydrolyzes extracellular TG, while hormone-sensitive lipase (HSL) hydrolyzes intracellular TG into FFAs [111]. Moreover, in conditions associated with insulin resistance, increased levels of FFAs are observed within the liver, leading to increased production of TG-rich lipoproteins, including VLDL [111]. Insulin resistance is also associated with reduced apo-B100 degradation and increased apo-CIII production, which both increase VLDL production [121]. Increased levels of apo-CIII, in the absence of a high-fat diet, result in increased liver FFA and decreased antioxidant capacity, increased expression of TNF-α and IL-1β, and decreased expression of the adiponectin receptor, all of which have been associated with the development of MASLD [122].

FFA accumulation is characteristically observed in patients with MASLD, characterized by increased VLDL expression, positively correlated with hepatic TG accumulation and insulin resistance, LDL lipid peroxidation, and reduced total antioxidant status [123]. VLDL expression is closely associated with MTP, which is expressed mainly in hepatocytes and enterocytes, and is elevated in patients with MASLD. Carnitine palmitoyl-transferase 1 controls the entry of FFAs into mitochondria and is regulated by the peroxisome proliferator-activated receptor (PPAR)-α [124]. Upregulated PPARα increases FFA oxidation, while insulin resistance is negatively correlated with PPARα gene expression [125]. Furthermore, a diet high in fatty acids, has been suggested to induce hepatic insulin resistance, resulting in increases in plasma TNF-α and IL-6, in apo-CIII-overexpressing mice, with an increase in apo-CIII playing a major role in liver inflammation and cell death in patients with MASLD [126,127]. Finally, insulin’s anti-lipolytic function is dysregulated in cases of insulin resistance, which may facilitate hepatic TG synthesis. Overall, the aforementioned mechanisms enhance liver inflammation, oxidative stress, and mitochondrial dysfunction, resulting in liver injury, and predisposing to the development of MASLD, and subsequently MASLD-associated HCC.

## 5. The Risk of HCC Development in Patients with HFE Mutations

**Figure 5:**
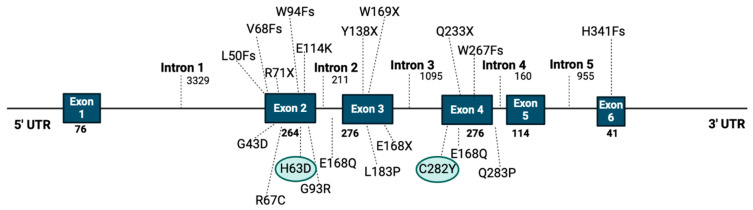
Schematic representation of the HFE gene demonstrating the location of the mutations, most of which located in exons 2 and 4. C282Y (exon 4) and H63D (exon 2) mutations are associated with a higher risk of HCC development in homozygotes (C282Y/C282Y homozygosity, C282Y/H63D compound homozygosity). (The image was created using BioRender software version 04, License # *LE27OL165H*).

Hereditary hemochromatosis (HH) is an autosomal recessive disease caused by a genetic alteration (variant or mutation) in genes that control iron metabolism [128]. Signs of IO include hepatic impairment and/or cirrhosis, joint pain, skin discoloration, heart failure, T2DM, sexual dysfunction, and, rarely, thyroid disease or HCC [128,129]. Three main mutations of the *HFE* gene have been currently described, with the most common *HFE* mutation being p.C282Y, which causes tyrosine replacement by cysteine in position 282 of the *HFE* gene [130]. Notably, p.C282Y homozygosity is observed in 0.3–0.6% of the European population [131]. The second most common mutation is p.H63D, which causes aspartame replacement by histidine [132]. p.H63D is present in 13.9% of the European population and is not always associated with IO, even in the homozygous state [132]. The global prevalence of HFE mutations is approximately 1.9% for p.C282Y, 8.1% for p.H63D, and 1.97% for compound homozygosity p.C282Y/H63D, while early diagnostic and therapeutic evaluation of HH can prevent disease complications and ensure a normal lifespan [129,133]. Therapeutic options involve regular phlebotomy or chelation treatment [134,135] while the majority of patients identified with *C282Y* homozygosity do not present significant end-organ damage [128]. However, recent cohort studies have demonstrated that patients (especially males) who are homozygotes for the *C282Y* mutation, and less frequently for the *C282Y/H63D* compound homozygosity, and present with serious and untreated IO, are at significant risk for cirrhosis and subsequently, HCC development (Figure 5) [136].

HCC is responsible for approximately 25–45% of disease-related premature deaths in patients with HH [137]. In 2007, Ellervik et al. performed a meta-analysis to address the incidence of HCC development in individuals carrying *C282Y* and *H63D* mutations [138]. The meta-analysis included nine studies and reported that *C282Y* homozygotes had a high risk for HCC development, with an odds ratio of 11 (3.7–34, I^2^ = 21%, 0–55%) for *C282Y* homozygotes [138]. Since then, several population-based studies investigating the correlation between *HFE* mutations and HCC have been conducted. In more detail, a study reported a significant correlation between HH and HCC, proving that decedents with hemochromatosis were 23 times more likely to have HCC than were decedents without hemochromatosis [139], while another study reported that the risk of HCC development in patients with HH is 20-fold higher than that of the general population [140]. Willis et al. reported that patients with HCC had a 7% prevalence of the *C282Y* homozygous mutation [141], while another study reported that, among 118 *C282Y* homozygotes, eight homozygotes were diagnosed with HCC, representing 1.8% of HCC patients [142]. A more recent meta-analysis with 43 included studies (5758 cases and 14,741 controls) demonstrated that the *HFE C282Y* homozygous mutation was significantly associated with increased risk for HCC, as compared to controls [143]. Another meta-analysis of nine studies (1102 cases and 3766 controls) also demonstrated a significant association between HCC and the *C282Y* polymorphism [136].

In patients with HH, the main risk factor for HCC development is the presence of cirrhosis [144]. Early studies suggested that HCC development in HH patients in the setting of cirrhosis was 200-fold higher, as compared to non-cirrhotic controls [145]. This result, however, was observed by studying populations within which HH diagnosis was based on clinical features and biochemical assays, and not on HFE genetic testing [144]. On the other hand, recent studies with HH being diagnosed using a combination of genetic testing, clinical examination, and abnormal iron accumulation as measured by serum iron concentration (transferrin saturation and serum ferritin) and by iron deposition in T2* MRI or liver biopsy have reported an approximately 20-fold higher risk of HCC development in cirrhotic HH patients, as compared to non-cirrhotic [140]. Moreover, a meta-analysis of eight studies demonstrated that the annual incidence of HCC in HH cohorts is 1.20% per year [138]. Notably, a recent study demonstrated a higher percentage of approximately 4%, while hepatic iron accumulation has been suggested to be directly linked to HCC development, independently of cirrhosis [146,147]. Chronic viral hepatitis, alcohol consumption, T2DM, increasing age, and male gender are other confounding risk factors for HCC development in HH patients, alongside cirrhosis [144]. Furthermore, serum ferritin level above 1000 mg/L at diagnosis, being indicative of clinically significant iron overload and predisposing to cirrhosis, has been recently indicated as a risk factor for HCC in patients with HH [148]. It has been also suggested that patients with cirrhosis and HCC are more likely to carry a *C282Y* mutation (HFE heterozygotes) than cirrhotic patients without HCC [149]. The incidence of both heterozygous *C282Y* and *H63D* mutations was also observed to be higher in patients with cirrhosis-associated HCC than in controls (8.6% vs. 1.6%) [142].

## 6. Hepcidin Expression in MASLD and HCC: Pathophysiological Correlation and Raising Enigmas

Hepcidin expression is regulated by both physiological modulators and pathological conditions, including neoplastic disease [150]. Physiological modulators include systemic and tissue iron levels (involving transferrin expression and the BMP/SMAD pathway), which are negatively correlated with hepcidin expression, inflammation (mostly IL-6 expression), erythropoiesis, hypoxia, which suppresses hepcidin expression via an erythropoietin-facilitated increase in erythropoiesis, growth factors (e.g., hepatocyte growth factor and epidermal growth factor) which decrease hepcidin expression, sex hormones (e.g., progesterone and testosterone), which are negatively correlated with hepcidin expression, and erythroid regulators like growth/differentiation factor-15 (GDF-15) and twisted gastrulation BMP signaling modulator-1 (TWSG1), which have been suggested to downregulate hepcidin expression [150]. Overall, the regulatory pathways of hepcidin expression are the BMP/SMAD pathway, the IL-6 pathway, and the erythropoietin–erythroferrone (EPO–ERFE) axis [151]. Liver disease plays a central role in the regulation of hepcidin expression. Considering the involvement of iron in advanced liver disease, including liver failure, cirrhosis, and HCC, the relationship between these conditions and the regulatory mechanisms of hepcidin is currently under investigation [150].

### 6.1. Hepcidin Expression in MASLD

Dysregulated iron metabolism is a primary disease-characteristic in patients with MASLD and an independent predictor of advanced liver fibrosis [150]. Systemic inflammation and chronic high-fat diet, characterizing MASLD, are both associated with increased iron concentration, which upregulates hepatic hepcidin production [152]. Increased hepcidin production is also a result of high glucose-stimulated pancreatic b-cell function [153]. The significant excess of iron and lipids, resulting from the activation of the inflammatory cascade and insulin resistance that dysregulates lipid metabolism, exacerbates liver damage, mainly by oxidative stress [154]. Therefore, serum hepcidin in patients with MASLD is higher compared to non-MASLD individuals, while the increase in serum hepcidin results in a decrease in ferroportin expression and cellular iron output, which further induces iron deposition in hepatocytes [154]. Patients with MASLD and IO present with higher grade of fibrosis and worse liver function test results [154]. It is also worth noting that the increased levels of hepcidin in MASLD patients are attributed to increased inflammatory cytokine expression, mainly IL-6 [152]. Hepcidin expression is a potential non-invasive biomarker of MASLD [154], while the exact mechanism by which hepcidin contributes to MASLD development is still being investigated. Many studies have associated liver IO with the occurrence of HCC in MASLD. Tsutsumi et al. reported a significant negative correlation between hepcidin immunoreactivity and fibrosis grade in pediatric population with MASLD, indicating the reduction in hepcidin in response to increased iron levels as a potential mechanism leading to liver fibrosis [155]. Finally, Sorrentino et al., analyzing a cohort of 153 patients with MASLD-related cirrhosis, suggested that liver iron deposition was of greater incidence and severity in patients with HCC as compared to controls [156].

### 6.2. Hepcidin Expression and Regulation in HCC

#### 6.2.1. Hepcidin Expression Patterns in HCC

Liver is the main site of hepcidin production; thus, HCC has been strongly associated with hepcidin dysregulation [157]. The normal range of serum hepcidin in the human body is between 2 and 20 nm, while hepcidin expression is mostly elevated in many types of cancer, including prostate cancer, multiple myeloma, breast cancer, and non-lymphoma Hodgkin’s disease [158,159]. Iron homeostasis is triggered by mutations in tumor cells or tissues and is further dysregulated by altered expression of hepcidin [143]. In cases of increased hepcidin levels, iron transfer from enterocytes and macrophages into the circulation is inhibited, while in cases of decreased hepcidin levels, plasma iron levels are upregulated, resulting in various levels of toxicity [160]. Elevated hepcidin levels lead to ferroportin downregulation, which results in overactivated signaling pathways, including the Wnt and NF-kB pathways, correlated with tumor progression.

In order to define whether serum hepcidin is a potential molecular target for HCC diagnosis, prognosis, and treatment, it is essential to compare hepcidin levels (serum and/or tissue) of HCC patients to healthy controls. Several studies have reported decreased serum hepcidin in HCC patients, as serum hepcidin levels have been proven to be decreased in non-viral HCC patients as compared to controls, and hepcidin expression in HCC biopsy specimens has been observed to be significantly lower compared to normal tissue, while HCC cell lines have shown lower hepcidin expression as compared with primary human hepatocytes, and mouse models of HCC have shown low hepcidin expression [161,162,163]. However, elevated hepcidin levels in HCC have been demonstrated by other studies, conducted in vitro or in silico. Characteristically, a study on hepcidin expression using GEO dataset GSE57957 reported that hepcidin was upregulated in HCC, as compared with surrounding healthy tissue [164,165]. Similarly, patients diagnosed with HBV-induced HCC presented with increased serum hepcidin levels as compared with healthy controls [166]. Some authors suggested that hepcidin expression was not significantly different between tumors at varying levels of differentiation, whereas others reported that hepcidin mRNA expression was much lower in patients with multiple HCC tumor metastatic lesions [166,167]. Kijima et al. reported that serum hepcidin concentrations did not significantly correlate with hepcidin mRNA expression in cancerous or non-cancerous tissue [167]. Specifically, while hepcidin mRNA expression was low, serum hepcidin levels in HCC were high in some patients and normal in others. The results of these studies are summarized in Table 1.

#### 6.2.2. Hepcidin Regulatory Mechanisms in HCC

Hepcidin’s expression in HCC is regulated by several mechanisms at genetic, epigenetic, and protein level. Downregulation of hepcidin encoding gene (*HAMP*) via increased DNA methylation on *HAMP* gene promoter has been observed in patients with HCC, explaining the decrease in hepcidin expression levels. *HAMP* downregulation occurs despite normal serum iron (131.4 ± 23.4 mg/dL) and normal [179.5 (14–232.9 ng/mL)] or high [414.4 (328.2–1121 ng/mL)] ferritin levels in some HCC patients or elevated levels of iron, ferritin, and transferrin saturation in the serum of HCC patients, as compared to healthy controls [162,167]. Dysregulation of iron-sensing in HCC is another feature associated with abnormal serum hepcidin levels. Specifically, the downregulation of TFR2 and HJV due to decreased mRNA stability leads to decreased hepcidin expression, and the downregulation of ALK2 in HCC cell lines could explain the decrease in hepcidin in these cell lines [162,167,170]. Maltriptase-2 is a negative regulator of hepcidin, cleaving HJV on hepatocytes, while the increased maltriptase-2 expression reported in HCC is correlated with downregulation of hepcidin expression or prevention of hepcidin upregulation in HCC [171,172]. RUNX3 is a tumor suppressor transcription factor that prevents tumor cell migration and metastasis [173]. Moreover, iron-induced elevation of BMP6 is mediated by RUNX3 [173]. In HCC, RUNX3 is inactivated due to hypermethylation, leading to BMP6 blockage and hepcidin diminished expression [174]. *TP53* gene encodes p53 protein, which is a tumor suppressor protein, and IO downregulates p53 expression [174]. P53 binds *HAMP* promoter and activates its transcription; thus, the downregulation of p53 decreases *HAMP* transcription [175]. TP53 in HCC is mutated, which leads to downregulation of p53 and could partially explain the decrease in hepcidin expression in HCC [175]. GDF-15 is overexpressed in HCC tissue and high serum levels of GDF-15 have been observed in some recent studies [176,177]. GDF-15 has been proven to suppress hepcidin expression in primary hepatocytes; however, its role in IO-related HCC needs to be further clarified in vivo [176]. Circular RNA circ_0004913 can regulate HCC progression, enhancing its migratory, proliferative, and invasive capacity [178]. Targeting microRNA-184, which in turn targets HAMP, creates a positive correlation between its expression and serum HAMP levels [178].

BMPs and IL-6 are the two main drivers of hepcidin expression in hepatocytes in response to tissue IO and inflammation [160]. BMP6 is the most important hepcidin driver in response to IO, while BMP9, BMP4, and BMP2 induce in vitro hepcidin transcription in HCC cells [168]. High BMP levels are associated with worse HCC prognosis [168]. Recent studies have demonstrated a significant elevation in BMP6 and BMP4 levels in HCC, which is associated with increased hepcidin expression [162]. Apart from IO, hepcidin has been associated with other HCC risk factors, namely cirrhosis and alcohol consumption [179]. A recent study provided evidence that hepcidin levels were negatively correlated with HCC severity, with decreased hepcidin levels being associated with poor overall survival and progression-free survival [180]. Moreover, hepcidin has demonstrated hepatoprotective effects via suppressing hepatic stellate cells, by inhibiting TGF-β-induced SMAD3 phosphorylation [181]. Thus, hepcidin downregulation in HCC has been associated with worse disease outcomes [181]. Overall, shedding further light on hepcidin’s regulatory role in HCC is important to identify new molecular targets that may be of aid to the existing strategies of HCC diagnosis, prognosis, and treatment.

## 7. The Promising Role of IO-Induced Cell Death in HCC Pathogenesis and Risk Stratification

### 7.1. Ferroptosis: A Brief Overview of Pathophysiology and Its Association with Liver Disease

Ferroptosis represents an iron-dependent non-apoptotic mode of cell death that is accompanied by a large amount of iron accumulation and lipid peroxidation [182]. The term “ferroptosis” was established in 2012 by Dixon et al., who studied the mechanism by which erastin, a small inhibitor of cystine-glutamate transport receptor, caused cell death to *RAS*-mutated tumor cells [183]. Ferroptosis-inducing factors can directly or indirectly downregulate glutathione peroxidase, resulting in a decrease in its antioxidant capacity and accumulation of lipid ROS, subsequently leading to oxidative cell death [184,185]. Ferroptosis does not have the morphological characteristics of typical necrosis (swelling of the cytoplasm and organelles, rupture of cell membranes), apoptosis (cell shrinkage, chromatin condensation, cytoskeleton disruption), or autophagy (formation of autophagic vacuoles) [186]. It occurs as reduced mitochondrial volume and vanishing of mitochondrial cristae, while the nucleus remains at normal size and the cell membrane remains intact. Biochemically, a combination of regulatory mechanisms induces intracellular glutathione (GSH) depletion and decreases glutathione peroxidase 4 (GPX4) activity, resulting in the inability of lipid peroxides to be metabolized by the GPX4-catalyzed reduction reaction, and in Fe2 + lipid oxidization in a Fenton-like manner, subsequently producing a large amount of ROS, which promotes ferroptotic cell death [185,187].

Ferroptosis drivers are divided into four categories. The first category includes erastin, which selectively kills tumor cells expressing ST and RAS^V12^, and targets voltage-dependent anion channels, leading to mitochondrial dysfunction [187]. The second category includes RSL3 and DPI7, which directly inhibit GPX4 activity [187]. The third category includes FIN56, which induces ferroptosis by GPX4 degradation or by depleting endogenous antioxidant coenzyme Q10 via binding to the enzyme squalene synthase [187]. The fourth category includes FINO2, an organic peroxide which has many features in common with artemisinin, causing ferroptosis due to a combined effect of the direct oxidation of labile iron and the inactivation of GPX4 [187]. Ferroptosis is characterized by an increase in intracellular LIP, which is associated with increased production of free radicals, increased lipid peroxidation, and IO-induced cell death [187]. Several authors suggest that cancer cells have increased iron requirements compared to normal cells and that the increased intracellular LIP sensitizes tumor cells to ferroptosis [188]. Recent investigation has proven that ferroptosis is associated with the pathophysiological mechanisms of numerous diseases, including solid tumors, nervous system diseases, blood malignancies, and kidney injury [186].

The liver plays an important role in iron homeostasis, while unregulated excess iron contributes to hepatic disease [186,189]. Ferroptosis has therefore become a hotspot of HCC research, with many investigators aiming to include ferroptosis as a diagnostic marker and as a primary constituent of therapeutic schemas [186]. IO-induced ferroptosis plays a crucial role in the liver, being associated with the development of numerous liver-related pathologies leading to acute liver disease, ferroptosis-induced liver injury, and chronic liver disease, such as MASLD and hereditary hemochromatosis, predisposing to HCC development. MASLD is associated with increased lipid peroxidation and iron accumulation in hepatocytes, which contribute to extensive inflammation and liver cell death [190]. Ferroptosis has been proven to aggravate inflammation in the early stages of MASLD [191]. Additional studies suggested that ferroptosis and lipid metabolic disorders play an essential role in the progression of MASLD, while hindering ferroptosis can significantly decrease the severity and propagation of MASLD [192,193]. Specifically, ferroptosis inhibitors, including deferiprone and Trolox, suppress inflammation and hepatic cell death at the initial stage of MASLD [189]. These findings highlight the role of ferroptosis in several liver diseases as a potential risk factor for the development of HCC. Future studies are necessary to identify the precise molecular mechanisms underlying ferroptosis and lipid metabolism in the pathogenesis of MASLD.

### 7.2. Ferroptosis in HCC: Its Role in Tumorigenesis, Prognostic Utilization, and Novel Risk Stratification Scoring Systems

Ferroptosis is considered to present a different role in HCC, compared to other forms of hepatic disease, mainly presenting with tumorigenic, tumor-proliferating, and metastatic effects [194,195]. In more detail, ferroptosis-induced macrophage polarization was suggested to enhance pancreatic cancer development by studying *Pdx1-Cre;Kras^G12D+^;Gpx4^−/−^* mouse models [196]. Similarly, *Gpx4* depletion in HCC cells induces the ferroptotic death of hepatocytes, creating a tumor-suppressive TME, upregulating immune checkpoints inhibitors, and infiltrating myeloid-derived suppressor cells [196], suggesting that ferroptotic damage-mediated and inflammation-associated immune suppression promotes hepatocarcinogenesis. Moreover, ACSL4, an important ferroptosis promoter, is overexpressed and has been associated with poor outcomes in patients with HCC [197]. *ACSL4* depletion in HCC cells effectively inhibits tumor cell proliferation, by disruption of the c-Myc-sterol regulatory element binding transcription factor 1 pathway [197]. In contrast, upregulated expression of *ACSL4* in HCC patients is associated with decreased patient survival [198]. Furthermore, a recent study has demonstrated that lipid peroxidation-derived molecules (e.g., γ-OHPdG), are potential promoters of hepatocarcinogenesis [198]. Along the same line, Guo et al. showed that the overexpressed SLC7A11 in HCC has been positively correlated with tumor progression and worse patient survival, whereas SLC7A11 suppression attenuated HCC cell proliferation [199]. These results suggest that downregulating the expression of genes and proteins that are associated with ferroptotic damage could have promising results in HCC prevention.

**Figure 6:**
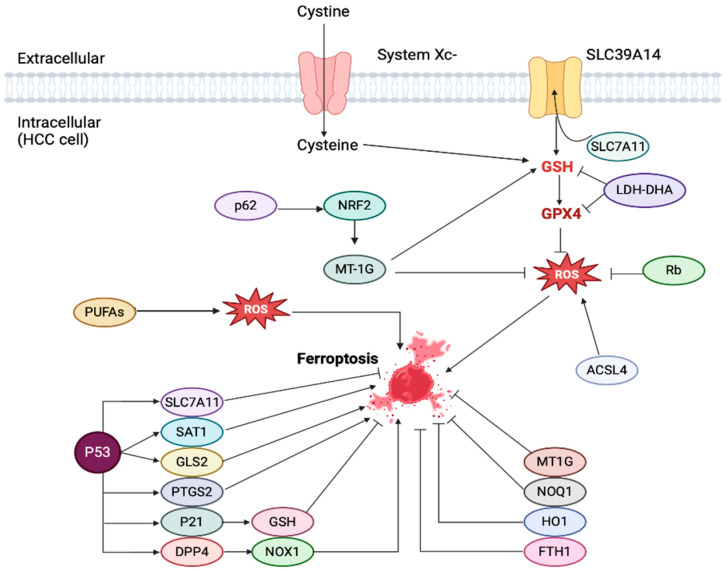
Regulators and pathways of ferroptosis in HCC. The initiators of ferroptosis include PUFA (lipid metabolism) and iron metabolism: SLC39A14 and extracellular-transported cysteine induce GSH expression, which inhibits ROS. *MT1G*, *NOQ1*, *HO1*, *FTH1*, *P62*, and Rb are also ferroptosis inhibitors, while ACSL enhances ROS formation. *SLC7A11*, *SAT1*, *GLS2*, *PTGS2*, *P21*, and *DPP4* regulated by p53 are the main ferroptosis-inducing genes. These observations could be the basis for a genetic-based risk stratification tool for ferroptosis-induced HCC. (The image was created using BioRender software version 04, License #*VP27OL1I2Y*). [HCC: hepatocellular carcinoma; ROS: reactive oxygen species; PUFA: polyunsaturated fatty acid].

The ferroptosis apoptotic pathway in HCC is regulated by numerous well-defined molecules in several stages (Figure 6). Specifically, the entrance of cyclin through the Xc^−^ system and its conversion to GSH are inhibited by erastin expression [200]. P53 is also an important regulator of the ferroptotic pathway by affecting the transcription level of several ferroptotic genes, including *SLC7A11*, *SAT1*, *GLS2*, *PTGS2*, *P21*, and *DPP4* [200]. RSL3 directly targets GPX4, while Rb and CISD1 are direct regulators of the intracellular ROS levels. GSTZ1 and P62 regulate ferroptosis by controlling Nrf2 entrance into the nucleus, which regulates the expression of the ferroptosis-enhancers: MT1G, NQO1, HO1, and FTH1 [200]. Another ferroptosis regulator and potential contributor of putative susceptibility of HCC cells towards ferroptotic cell death are ω-3 polyunsaturated fatty acids (PUFAs). Lim et al. demonstrated that PUFAs may reduce HCC growth by inhibiting the expression of HCC promoters: β-catenin and cyclooxygenase-2 (COX-2) [201]. Weylandt et al. suggested that PUFAs consumed in the form of fish that contain unsaturated fatty acids (n-3 PUFAs) can reduce the risk of HCC development [202]. It is clear that IO and ferroptosis regulators are promising key players, suggesting that HCC is a potential target for ferroptosis-based therapies [201,202]. Ferroptosis regulators analyzed herein are important in eliminating oxidative stress and tumor cell growth and are being overexpressed in HCC cells, and therefore, ferroptosis induction, via their pharmacological inhibition, might hold promise in HCC treatment [203].

In silico analysis has demonstrated a potential clinical correlation between ferroptosis-related genes and HCC prognosis [204]. A novel risk stratification tool has been established based on the expression of 25 ferroptosis-related genes in public TCGA, CGGA, and GEO databases, which were divided into three distinct clusters based on the genomic analysis of 371 HCC cancer samples [204]. The expression of *HSPA5*, *EMC2*, *SLC7A11*, *HSPB1*, *GPX4*, *FANCD2*, *CISD1*, *FDFT1*, *SLC1A5*, *TFRC*, *RPL8*, *DPP4*, *CS*, *CARS1*, *ATP5MC3*, *ALOX15*, *ACSL4*, and *ATL1* was significantly higher in HCC tissue compared with normal liver tissue, while the expression of *NFE2L2*, *MT1G*, *SAT1*, and *GLS2* was decreased in HCC tissue compared with normal liver tissue [204]. Survival analysis proved that the increased expression of *SLC7A11*, *SLC1A5*, *TFRC*, *RPL8*, and *CARS1* was associated with unfavorable overall survival in patients with HCC [204]. Moreover, another study investigated 374 HCC tumor samples using 214 ferroptosis-related genes from the FerrDb database [205]. This study suggested a four-gene (*GPX2*, *MT3*, *PRDX1*, *SRXN1)* overall survival-prediction tool for HCC patients [205]. PRDX1 was the hub gene of this prediction model and was highly expressed in HCC tissue [205]. Another study assessed 104 ferroptosis- and iron metabolism-related genes and proposed a risk stratification model consisting of four genes (*ABCB6*, *FLVCR1*, *SLC48A1*, and *SLC7A11*) for predicting HCC prognosis, classifying patients into high and low ferroptosis score groups [206]. Further in silico analyses of larger databases may lead to the development of a holistic ferroptosis-related risk stratification tool, with potential applicability in all HCC patients, regardless of individual tumor characteristics. The main ferroptosis regulators and signaling pathways in HCC are illustrated in Figure 6.

## 8. Iron Metabolism as a Novel Therapeutic Target for HCC

The potential role of iron metabolism as a novel therapeutic target for HCC consists of three aspects: (I) direct iron depletion, (II) modulating hepcidin expression, and (III) targeting ferroptosis. Recent studies have investigated whether iron depletion therapy, either phlebotomy or iron chelation, may successfully reduce the risk of hepatocarcinogenesis. To our knowledge, the only available prospective in vivo study on HBV-cirrhotic patients who underwent phlebotomy reported a decreased risk of HCC development, but this study lacked longitudinal patient monitoring [207]. Iron depletion has also been investigated as an alternative option to antiviral therapy. Phlebotomy (500 mL per week) after being tested as an alternative to interferon (IFN) to eight HCC patients who demonstrated poor response to interferon therapy, showed promising results and was suggested to improve liver biochemistry [208]. Along the same line, another study including 25 patients under iron depletion therapy for 5 years demonstrated that the fibrosis score significantly decreased in the treatment group, from 2.3 to 1.7 [209]. A strong correlation between the baseline level of liver biochemistry and its improvement after treatment was also observed in patients with high baseline serum ferritin levels [209]. Regarding MASLD patients, treatment with phlebotomy has been reported to improve insulin resistance, evaluated by fasting serum glucose, insulin levels, and the homeostatic model assessment-insulin resistance (HOMA-IR) score [208]. Moreover, two randomized controlled trials investigated the efficacy of iron reduction therapy in MASLD patients. The first trial was conducted in Italy and included 38 MASLD patients who were randomized to phlebotomy treatment or control group, undergoing liver biopsy before and after treatment. Disease severity improved in eight out of 12 patients in the treatment group, and in two out of nine patients in the control group [208]. The second trial was conducted in Australia and included 74 MASLD patients diagnosed by abdominal ultrasonography, many of whom presented with normal serum ferritin levels. This study did not show statistically significant results in insulin resistance and improvement in disease severity after the use of iron depletion therapy [208].

Iron chelation therapy has been a promising alternative as a potentiator of HCC therapeutics. Specifically, according to the results of in vitro studies, deferasirox is a potential enhancer of the tumor-inhibitory effect of sorafenib, suggesting cell cycle arrest and hepatic cell apoptosis as the main pathophysiological mechanisms [210,211]. Deferasirox inhibits cancer cell proliferation mainly by arresting cell cycle, and secondly by inducing apoptotic signaling pathways [210,211,212]. However, using sorafenib and deferasirox as monotherapy did not show favorable results in HCC treatment, while the combination schema of deferasirox and sorafenib synergistically inhibited cancer cell proliferation, inducing apoptosis in HepG2 cells, inhibiting cyclin-dependent kinase inhibitor p21 signals, which are capable of cellular repair, subsequently inhibiting cell death [210,211,212]. Another in vitro study demonstrated that deferasirox could induce apoptosis, reducing the proliferation of hepatoma cells lines, and thus suppressing HCC development [213]. The use of deferasirox resulted in a sharp elevation of *HAMP* mRNA expression, both in HCC and healthy tissue [213]. Therefore, apart from its role in iron depletion, deferasirox could be used to maintain hepcidin expression at normal levels and to regulate iron homoeostasis in HCC, while additionally exerting tumor-suppressive effects [213]. However, the introduction of chelation therapy in chemotherapeutic schemas of HCC needs further research, particularly due to concerns about dose-dependent toxicity [213].

Modulation of hepcidin expression in patients with HCC is a newly described potential therapeutic approach associated with promising results regarding patient survival. Various techniques inhibiting transcription are currently available in order to reduce enhanced hepcidin synthesis in various tumor subtypes [214]. Regarding hepatocarcinogenesis, research has focused on inhibiting the interaction between hepcidin and ferroportin, which leads to suppression of hepcidin activity [214]. Anti-HJV antibodies can arrest hepcidin expression, being part of local anti-hepcidin therapeutic combination schemas [214]. One novel hepcidin-oriented treatment strategy is using RNA-interference agents which block hepatic hepcidin, demonstrating relative effectiveness in recently conducted clinical trials [215]. Inhibiting the activity of regulators of hepcidin expression, mainly BMP molecules (BMPR, HJV, and maltriptase-2), is another important aspect of hepcidin antagonism [216]. The BMP/SMAD pathway can be affected using heparins, which downregulate hepcidin expression. Chemically synthesized heparins with non-anticoagulant characteristics have shown effectiveness in multiple myeloma and sarcoma, and are considered to be effective in suppressing tumor progression, given as monotherapy or in combination with conventional therapy [216]. The only possible use of heparins in HCC is in the case of chronic anemia associated with advanced HCC, characterized by high blood hepcidin, despite the fact that in most cases of HCC, hepcidin levels decrease [217].

Targeting ferroptosis is another potential therapeutic mechanism for the suppression of hepatocarcinogenesis. The classic chemotherapeutic medication, cisplatin, directly binds to GSH, creating a complex that inhibits GSH activation and enhances ferroptosis [218]. The combinatorial schema of cisplatin–erastin enhances the synthesis of diphenylethylene dichloride, consuming intracellular glutathione, inducing ferroptosis [219]. Additional medication, namely sulfasalazine and buthionine sulfoximine, indirectly deplete glutathione, activating the ferroptosis pathway [220]. Furthermore, recent investigation has proposed radiation therapy as an inducer of ROS generation, triggering the upregulation of SLC7A11 while suppressing ferroptosis [221]. Yuan et al. discovered that cluster element (CLTRN) acts as a radiosensitive locus, potentially enhancing HCC radiosensitivity by modulating ferroptosis via the glutathione metabolism pathway, and suggested CLTRN as a promising target for radiotherapy in HCC [222]. Newly studied ferroptosis inhibitors are currently considered a beneficial therapeutic option for the treatment of HCC. To begin with, the intracellular antioxidant coenzyme Q10 (CoQ10), which modulates ferroptosis via regulating the level of OS and antioxidant enzyme activity, is suggested to be a promising approach for HCC ferroptosis-targeting therapeutic evaluation [223]. Ferrostatin-1 (Fer-1), one of the first class of synthetic rosanotriterpene A (RTA) compounds inhibiting ferroptosis, as well as liproxtatin-1 (Lip-1) and liproxtatin-2 (Lip-2), which are iron sag inhibitors promoting sequential reactions including ferritin degradation, lipid peroxidation, and subsequent ferroptosis, are also suggested as HCC therapeutic options in recent studies [224]. The use of long non-coding RNA (LncRNA) transcripts has shown contradictory results; HCG18 has been proven to modulate GPX4 leading to ferroptosis of HCC cells by targeting microRNAs and to increase sorafenib sensitivity, while the knockdown of LncRNA SNHG1 and LncRNA GABPB1-AS1, targeting genes associated with ferroptosis, has been shown to increase iron metabolism accumulation and lipid peroxidation, leading to HCC progression [225,226]. Regarding investigation on the role of microRNAs on ferroptosis targeting therapy, MiRNA-214-3p enhances GPX4 protein stability and upregulation of SLC7A11 expression by inhibiting activating transcription factor 4 in HCC, which induces ferroptosis and suppresses hepatocarcinogenesis, and MiR-612 affects HCC oncogenic properties by downregulating coenzyme Q10 and increasing intracellular PUFA and lipid peroxidation processes [227].

Finally, IO-targeting nanotechnology-based therapeutic approaches have shown promising results in HCC treatment. The use of LDL-docosahexaenoic acid (DHA) nanoparticles as a part of rat and human liver cancer cells, and their involvement in ferroptotic cell death, and the recently proposed use of nanobubbles in combination with oxygen-enhanced sonodynamic therapy for the treatment of HCC through ferroptosis, expectedly gathered significant attention [228]. Nanotechnology-based ligands have been shown to enhance HCC chemosensitivity by specifically interacting with HCC tissue surface receptors, being a potential part of novel HCC targeted therapeutic schemas. Characteristically, the antitumor-drug nanocarrier graphdiyne oxide (GDYO) has been suggested to promote endothelial HCC cell proliferation via receptor-mediated lysis and to be involved in the release of sorafenib, providing chemotherapeutic effects, and SLC7A11, inducing cancer cells to enter the iron cycle [229,230]. CE-Gal-NPs represents a small molecule nanomedicine system that induces ROS-mediated ferroptosis by targeting HCC by using the ligand galactose (Gal) by a nanotechnology-based system, which also upregulates the level of lipid peroxidation in both healthy and tumor cells, serving anti-tumor effects [231]. MIL-101(Fe) NPs, a drug-loaded nanoparticle ligand designed for HCC treatment, has been proven to significantly induce ferroptosis in HCC cells, mainly by increasing lipid peroxidation and malondialdehyde levels [232], while MIL-100@Apa@MPN, a metal-organic framework drug delivery system with remarkable thermal stability, is a potential anti-HCC therapeutic component, by targeting the ferroptosis pathway [233]. The aforementioned findings present promising and potentially revolutionary treatment options; however, further investigation is required to translate these observations into clinical practice.

## 9. Discussion and Future Perspectives

HCC is a major global health challenge, but despite scientific advances and implementation of novel diagnostic tools for the early detection of HCC in high-risk populations, patient survival has barely improved during the last three decades. Epidemiological changes in the causes of death worldwide due to scientific advances and increased life expectancy have added several levels of complexity to the landscape of HCC, in terms of early diagnosis and risk stratification. Specifically, HCC is becoming the main cause of death in chronic patients, for whom advances in treatment have increased their life expectancy at the expense of advanced liver damage, while metabolic syndrome is becoming the main risk factor for HCC development, especially in Western countries [234,235]. In recent years, promising targeted therapies have emerged as integral components of primary liver cancer treatment across all disease stages and age groups [236,237]. Notably, therapies targeting MASLD have shown potential to effectively disrupt the progression of tumor development, offering a new avenue for intervention [237]. Currently, most HCC staging systems and therapeutic schemas are based on tumor mutation burden and disease staging [238]. Due to the complexity of HCC responses to immunotherapy, it is not easy to define the adverse biological characteristics that affect sensitivity to chemotherapy and patient survival [238]. Given the high levels of serum ferritin that have been observed in many oncologic cohorts and the inclusion of serum ferritin as a part of cancer prognostic scores, IO has been studied in many types of tumors, including HCC, and is suggested to alter the immune TME, and to promote proliferative, invasive, and migrating capacity of tumor cells [239]. Thus, iron metabolism has significant potential as a therapeutic target for HCC and has become a focal point of ongoing research efforts worldwide.

IO is suggested to be an important risk factor for hepatocarcinogenesis. Iron excess on hepatocytes promotes ROS formation, which subsequently leads to the activation of oncogenic transcription molecules and signaling pathways [50]. Considering the increased incidence of MASLD-related HCC worldwide, recent investigation has focused on iron’s involvement in dysregulated glucose and lipid metabolism, as an aggravating factor of insulin resistance and disturbed liver biochemistry [240]. The markedly higher incidence of HCC in patients with hereditary hemochromatosis compared to the general population, along with well-established risk factors for hepatocarcinogenesis in the context of hereditary iron overload states, such as cirrhosis, diabetes, age, and male sex, represents a critical aspect of studying HCC pathogenesis. These insights could pave the way for the development of tailored screening protocols for patients with rare IO-related genetic disorders [143,240]. The potential hepatocarcinogenic role of hepcidin dysregulation and ferroptotic activity in cases of IO is considered to be a promising hallmark of therapeutic investigation in the near future [168,203]. It remains unclear whether hepatic iron accumulation directly acts as a primary driver of hepatocarcinogenesis in some cases. However, it is well-established that iron overload (IO) serves as a potent co-carcinogenic factor. When combined with established pathogenetic mechanisms of HCC, IO contributes to an increased mutational burden, ultimately facilitating cancer development. Consequently, regulating cellular and systemic iron concentrations may play a pivotal role in preventing HCC or delaying its progression. Given the limited understanding of the role of IO in hepatocarcinogenesis, the intricacies of iron homeostasis, and the dysregulation of iron metabolic pathways, ongoing research presents new opportunities. These challenges aim to advance our knowledge of HCC genetics and pathogenesis and promote the development of novel diagnostic, prognostic, and therapeutic strategies centered on IO modulation.

Despite promising investigation on the role of iron metabolism in HCC diagnosis and therapy, several limitations and challenges persist in current research. The diversity and heterogeneity of studied factors, spanning genetic, epigenetic, and metabolic mechanisms, and the variability in patient cohorts (e.g., cirrhotic versus non-cirrhotic HCC, HBV/HCV-related versus non-viral HCC), and the presence or absence of inherited iron overload diseases limit the reliability and comparability of results. Additionally, clinical guidelines for diagnostic evaluation and prognosis require an update to incorporate the classification and staging of iron metabolism disorders, particularly following the inclusion of hepcidin as a central biomarker. While the hepcidin and ferroptosis pathways show potential, most evidence arises from in vitro studies on cell lines or in vivo animal models. Consequently, their specificity and sensitivity as diagnostic and prognostic markers for HCC remain key areas for further research. Notably, many observations discussed in this review are derived from gene knockout or overexpression techniques. Such approaches may not fully capture the authentic physiological and pathological roles of hepcidin and ferroptosis in human models, highlighting the need for further studies that better reflect real-world human biology. Another important limitation of current research is that the studies regarding the incidence of HCC in cirrhotic HH patients, include populations with a genetic diagnosis of HH late in its course, despite the fact that genetic testing is widely available in most countries [241]. These patients underwent extensive, time-consuming imaging evaluations, which contributed to HCC progression and the development of severe clinical manifestations by the time of diagnosis. As a result, these cohorts were often ineligible for the initiation of novel iron-overload (IO)-related therapeutic options due to advanced disease stages. Moreover, most of the available studies were conducted in Caucasian and European populations, where the incidence of hereditary hemochromatosis (HH) is higher than the global median. This demographic bias raises concerns about the generalizability and applicability of these findings to patient cohorts with differing ethnic and geographic backgrounds. The identification of important molecules and regulatory mechanisms for iron metabolism and homeostasis during the last two decades has potentiated the understanding of the close correlations between dysregulated iron metabolism and HCC tumorigenesis and progression. Investigating the mechanisms by which iron triggers hepatocarcinogenesis has led to extensive efforts of developing IO-related targeted interventions for advanced HCC patients, which could be introduced in novel HCC immunotherapeutic schemas. On the contrary, there are certain aspects regarding the mechanism associating iron accumulation with hepatocarcinogenesis (e.g., mitochondrial iron accumulation, IL-6-controlled hepcidin expression) that are incompletely clarified.

Thus, current progress regarding the pathogenetic mechanisms controlling iron excess-related hepatocarcinogenesis has revealed several topics that need further investigation. Developing more precise and accurate methods to investigate the complex function of mitochondrial mRNAs, for instance, utilizing single-cell sequencing technology, could be of great importance for a deeper knowledge of the IO-related oncogenic mechanisms of oxidative stress [242]. To advance the field, it is essential to conduct additional preclinical and clinical studies to validate the safety and efficacy of novel therapeutic targets. Expanding databases such as TCGA, CGGA, and GEO is also crucial for enabling robust in silico analyses and enriching findings from animal and human models. Although significant knowledge gaps remain, it is well-established that iron plays an indispensable role in cancer development and progression. Based on this understanding, deeper mechanistic insights into the relationship between iron and HCC pathogenesis are anticipated to drive the development of more effective risk stratification tools and therapeutic strategies for HCC.

## 10. Conclusions

Iron overload plays a multifaceted oncogenic role in hepatocarcinogenesis, encompassing genetic, epigenetic, metabolic, and apoptosis-related mechanisms, while also being deeply implicated in the pathogenesis of MASLD. Despite extensive research, it remains uncertain whether iron-targeting therapeutics can significantly improve survival outcomes in patients with advanced HCC. Given the rising global mortality rate of HCC and the high prevalence of the metabolic syndrome that contributes to the increasing incidence of MASLD-associated HCC, it is imperative to prioritize the investigation of the role of iron metabolism in HCC progression, particularly in the context of metabolic syndrome. Future research should focus on key areas, including the BMP/SMAD signaling pathway and its regulation by hepcidin, the function and localization of IO-related transcription factors, and the modulation of ferroptosis. These areas, which have produced many novel findings, hold promise for advancing our understanding of IO-related mechanisms and their application in the development of diagnostic tools and therapeutic strategies for HCC.

## Figures and Tables

**Table 1:** Hepcidin expression levels in HCC tissue or serum as measured in independent patient cohorts with different disease characteristics. Most studies have demonstrated decreased hepcidin levels, while few of them showed an increase in hepcidin expression in non-viral HCC patients. The biological material used (serum or tissue) for hepcidin measurement in each study is marked with (+). HCC type regarding the presence of viral hepatitis is depicted in the table with (+) under each category. [HBV: hepatitis B virus; HCV: hepatitis C virus; REF: reference].

REF	Serum	Tissue	HBV-HCC	Non-Viral HCC	Median	Range/SD	Control	Hepcidin Level (Increased/Decreased)
[168]	+			+	4.62 nm	3.28–6.51 nm	4.33 to 8.41 nm	Normal
[161]	+		+		200 ng/mL	n/a	600 ng/mL	Decreased
[162]	+			+	175 ng/mL	±175 ng/mL	250–550 ng/mL	Decreased
[163]		+		+	2351 copies/mL	±505 copies/mL	16,308 ± 2194 copies/mL	Decreased
[169]	+		+		9.3 ng/mL	±4.9 ng/mL	4.8 ± 2.0 ng/ml	Increased
[167]	+			+	(1) 42.6 (2) 15.5	(1) 35.6–75.0 (2) 1.2–28.5	22.2 ± 12.3 ng/ml	Increased (1)/Normal (2)

## Data Availability

Not applicable.
